# The Territorial R.A.M.C. in London

**Published:** 1909-03-13

**Authors:** 


### The Hospital

A Journal of

The Medical Sciences and Hospital Administration.

New Series. No. 106, Vol. IV. [No. 1178, Vol. XLV.] SATURDAY, MARCH 13, 1909.

### THE TERRITORIAL R.A.M.C. IN LONDON.

Unsatisfactory as was the condition of many of the units of the Territorial Force a month ago in point of numbers, there were few branches of it in which so little progress had been made as in the R.A.M.C.T. Since Lord Esher's appeal to the daily Press, and the phenomenal response thereto, this reproach is in a fair way to be removed, at least as far as numbers are concerned. A month ago the London Mounted Brigade Field Ambulance had not only not obtained "recognition," but had no existence at all except on paper. It had the names of a commanding officer and the requisite complement of junior officers —in a pigeon-hole at the War Office—but these gentlemen were not definitely commissioned to this unit, and were still doing duty with their old regiments and battalions. It had no rank and file, no staff, no sergeant-major, no equipment, and no headquarters; in fact, its existence was totally unreal. Now at least it has recruited to full strength, and it is to be noted that it now automatically becomes the province of the County Association to provide headquarters and equipment out of the very meagre grants which Mr. Haldane or his advisers allow for this purpose. In the same way the fourth and fifth (Infantry Division) Field Ambulances have taken advantage of the boom in martial ardour, and are recruiting steadily towards full strength, if indeed they have not already attained it by this time.

It is no part of our province to discuss here the methods by which the success of Lord Esher's appeal has been obtained. The published letter which he has addressed to the London commanding officers, and the appreciative comments of the *Times* in its leading article of February 25 represent, of course, the natural gratification of those who, having won, can laugh. But it may not be out of place to hint, without the slightest intention of disparaging the work that has been done in so electrical a fashion, that they laugh best who laugh last; and that bricks cannot be made without straw. We wish to point out to the War Office, the County Associations, the profession, and the public, that men cannot be trained into efficient members of an ambulance corps unless adequate headquarters, equipment, etc., are provided. Now the problem of how this is to be done in London is admittedly not an easy one; but everyone who is conversant with the facts knows that the attempts to tackle it hitherto have been of the feeblest. Under the provisions of the Territorial Act the old headquarters of the already existing Volunteer Companies of R.A.M.C. at Calthorpe Street and Woolwich respectively were taken over by the County Association, just as were those of the various Infantry battalions. But there is this fundamental difference between the two cases of the Territorial R.A.M.C. and the Infantry: the latter are old regiments with drill-halls, headquarters, and so forth, already provided out of subscriptions and Governmental grants in Volunteer days; whereas many of the former are new conceptions entirely, inheriting from no predecessors either men, equipment, or accommodation of any sort. Yet, if we read the Territorial Regulations aright, no financial provision is made to enable County Associations to give the new units a fair start, and certainly those bodies have not so far made any practical effort to get over the difficulty. Hitherto all that has been done is to allot to the two already existing buildings—erected at Volunteer expense in the old days—ambulances and general hospitals far in excess of anything there is accommodation for; and if this plan is repeated in connection with the three newly recruited units, the congestion will be worse than ever.

Apart altogether from the question of proper efficiency, which is obviously unattainable unless ample room for storage of equipment, for drill, and for instruction is provided, there are other considerations which must not be left out of the reckoning. It is no use blinking the fact that ambulance work never has attracted the best and smartest young fellows of the type that fill our Yeomanry regiments and the crack Infantry corps, nor is it to be supposed that it ever quite will do. It is the opinion of all who have seen the men who have flocked to the colours lately that in point of physique their general average is extremely high; and this applies also to the recruits obtained by the various ambulances. Now if these youths and young men are to be put to the best use—that is, thoroughly trained for their duties and imbued with an enthusiasm for their corps which will impel them to introduce their friends of equally good standing to fill up the gaps due to resignations—it is essential that their headquarters should be made attractive both by the due provision of facilities for recreation and physical development, and by making possible a high level of efficiency. If men whose daily work is in an office are to be expected to drive a wagon and team of horses on Salisbury Plain during the summer camp, they must be afforded chances of practical instruction during the drill season; and that instruction must be, and of right ought to be, gratuitous. With the extensive field equipment of these ambulances we assert with conviction that the question of keeping in order the various wagons and gear, the proper care of harness, saddlery, and other equipment, the intelligent management of the draught horses, their shoeing, feeding, and attention generally—all this ought to come even before instruction in the care of the sick. For an ambulance unit which cannot transport itself to the arena of battle is not of much use to the wounded, however carefully its members have been taught to bind up wounds. Now the latter duties can be, and no doubt will be, very efficiently taught at schools of military instruction and by the officers of the units. What we wish especially to emphasise is the paramount importance of the other parts of the training and the impossibility of a high standard therein unless the County Associations begin to show a very different spirit.

